# Supplementary material for: Assessment of Crosslinkers between Peptide Antigen and Carrier Protein for Fusion Peptide-Directed Vaccines against HIV-1
Source: Vaccines (Basel). 2022 Nov 12;10(11):1916. doi: 10.3390/vaccines10111916 (PMC9698951; doi:10.3390/vaccines10111916)
Supplement: Supplementary file 1 [file vaccines-10-01916-s001.zip › vaccines-1985631-supplementary.pdf]

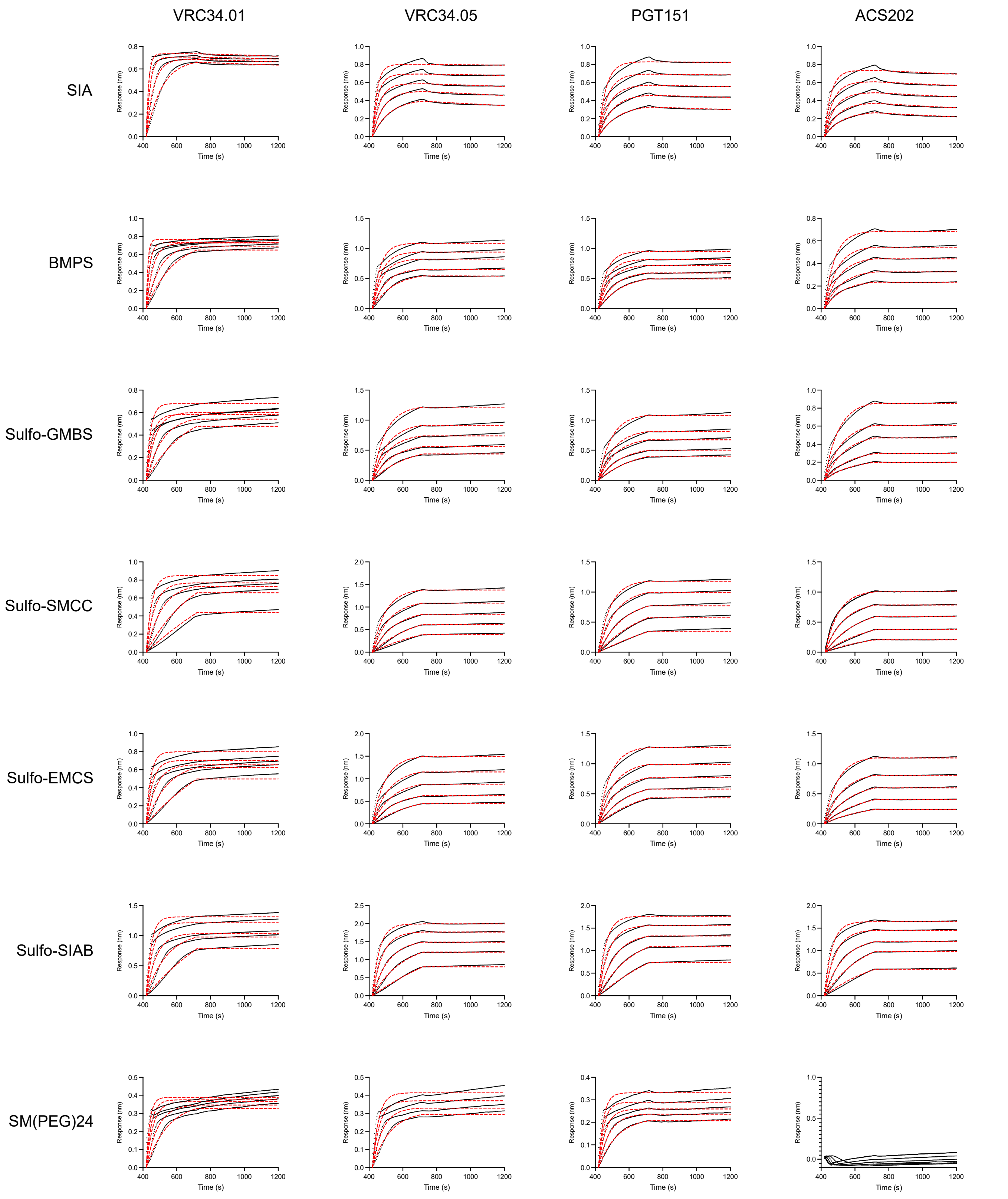

Figure S1. BLI binding curves for antigenicity measurements of the conjugates.
Binding curves are shown as black lines, while red lines indicate the global fits generated by using a 1:1 Langmuir binding model.

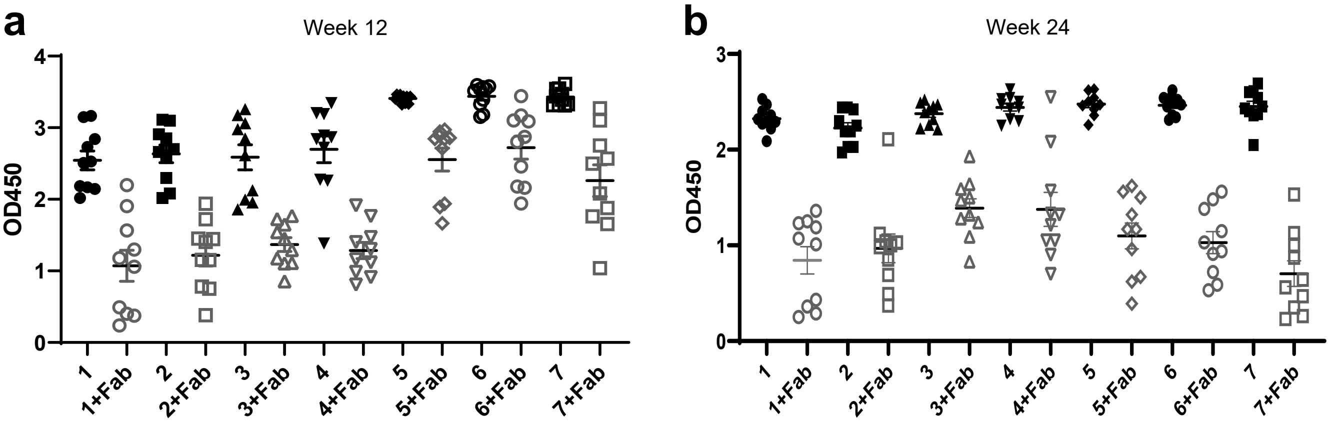

Figure S2. Env trimer anti-base responses.
Anti-base responses were measured using the antigen-binding fragment (Fab) of base-directed antibody R19R in a competition assay at wk12 (**a**) and at wk24 (**b**). Solid and empty symbols represent OD450 values of serum at 1:500 dilution without and with R19R competitor with BG505 DS-SOSIP trimer.

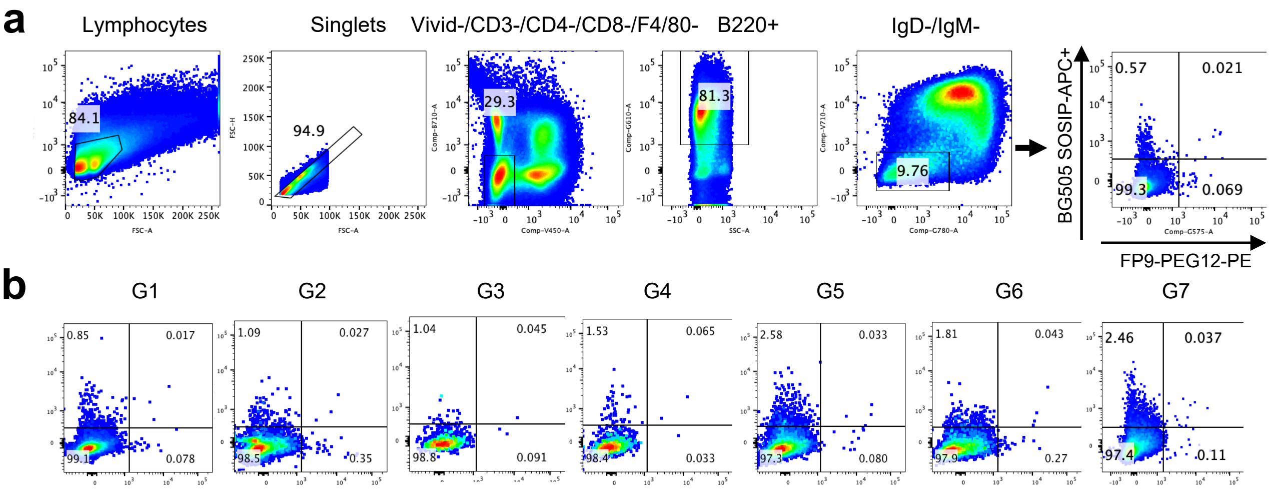

Figure S3. Double positive B cell quantification.
(**a**) Gating strategy for antigen-specific B cell FACS analysis. Splenocytes from a naïve mouse were used as a control to set up gating for the analysis of splenocytes from immunized mice. (**b**) Representatives with median values of FP+BG505+ B cell frequency for each group are shown for 7 groups.

p value:

<= 0.0005
0.005 - 0.0005
0.05 - 0.005
> 0.05

R^2:

>= 0.8

Serological properties

Linker and conjugate properties

	FP Octet responses (Week 4)	FP Octet responses (Week 6)	FP Octet responses (Week 24)	Trimer ELISA (Week 6)	Trimer ELISA (Week 8)	Trimer ELISA (Week 10)	Trimer ELISA (Week 24)	Trimer base response (Week 12)	Trimer base response (Week 24)	Double positive B cells (%)
Spacer length (Å)	0.07	0.14#	-0.14	0.14	-0.12	-0.07	0.71	-0.86#	0.36	-0.07
Molecular weight	0.29	0.09#	-0.02	0.24	-0.01	-0.10	0.47	-0.81	0.14	0.21
Stoichiometry	-0.01	-0.46#	0.05	0.00	0.04	0.16	0.33	0.13	-0.20	-0.18
Multimerization	0.70	0.77	0.25	0.48	0.31	0.08	-0.69	-0.20	-0.33	0.69
LogP	0.57	0.94#	0.29	0.50	0.35	0.50	-0.50	-0.24	-0.14	0.25
SH reactive group	0.13	-0.65	0.13	0.00	0.19	0.25	0.44	0.00	-0.38	-0.47
Ag. score	0.59	0.65	0.48	0.59	0.49	0.65	-0.11	-0.03	-0.48	0.00
RU (VRC34.01)	0.51	0.82	0.15	0.46	0.12	0.39	-0.10	-0.34#	-0.10	0.41
kon (VRC34.01)	0.21	-0.03	0.52	0.36	0.49	0.57	0.08	0.36	-0.52	-0.43
RU (VRC34.05)	0.93#	0.61	0.60	0.72	0.70	0.55	-0.25	-0.25	-0.74	0.13
kon (VRC34.05)	-0.37	0.90	-0.28	-0.16	-0.30	0.29	-0.22	0.11	0.60#	-0.20
RU (PGT151)	0.93#	0.89	0.61	0.84#	0.65	0.46	-0.25	-0.38	-0.59	0.43
kon (PGT151)	-0.14	-0.81	0.04	-0.30	0.02	-0.20	0.18	0.34#	-0.35	-0.23
RU (ACS202)	0.80#	0.90	0.71	0.80#	0.70	0.55	-0.36	-0.12	-0.60	0.31
kon (ACS202)	-0.25	0.80	-0.50	-0.21	-0.34	0.11	-0.04	-0.43	0.79#	-0.29

Figure S4. Correlations between linker and serological properties.
Spearman rho values are shown. Correlations with high Spearman significance ($p <= 0.05$) are highlighted in purple (scale shown). Correlations which maintained Spearman significance when outlier SM(PEG)24 was excluded are denoted with a #. Correlations which failed to meet requirements for Spearman significance and Pearson correlation assumptions but fit to a linear (or log-transformed linear) model ($R^2 >= 0.8$) are highlighted in red. For serological properties, the geometric mean was taken over each participant group, with 0 values converted to 1. For the SH reactive linker property, Iodoacetyl was coded as 0 and Maleimide was coded as 1.

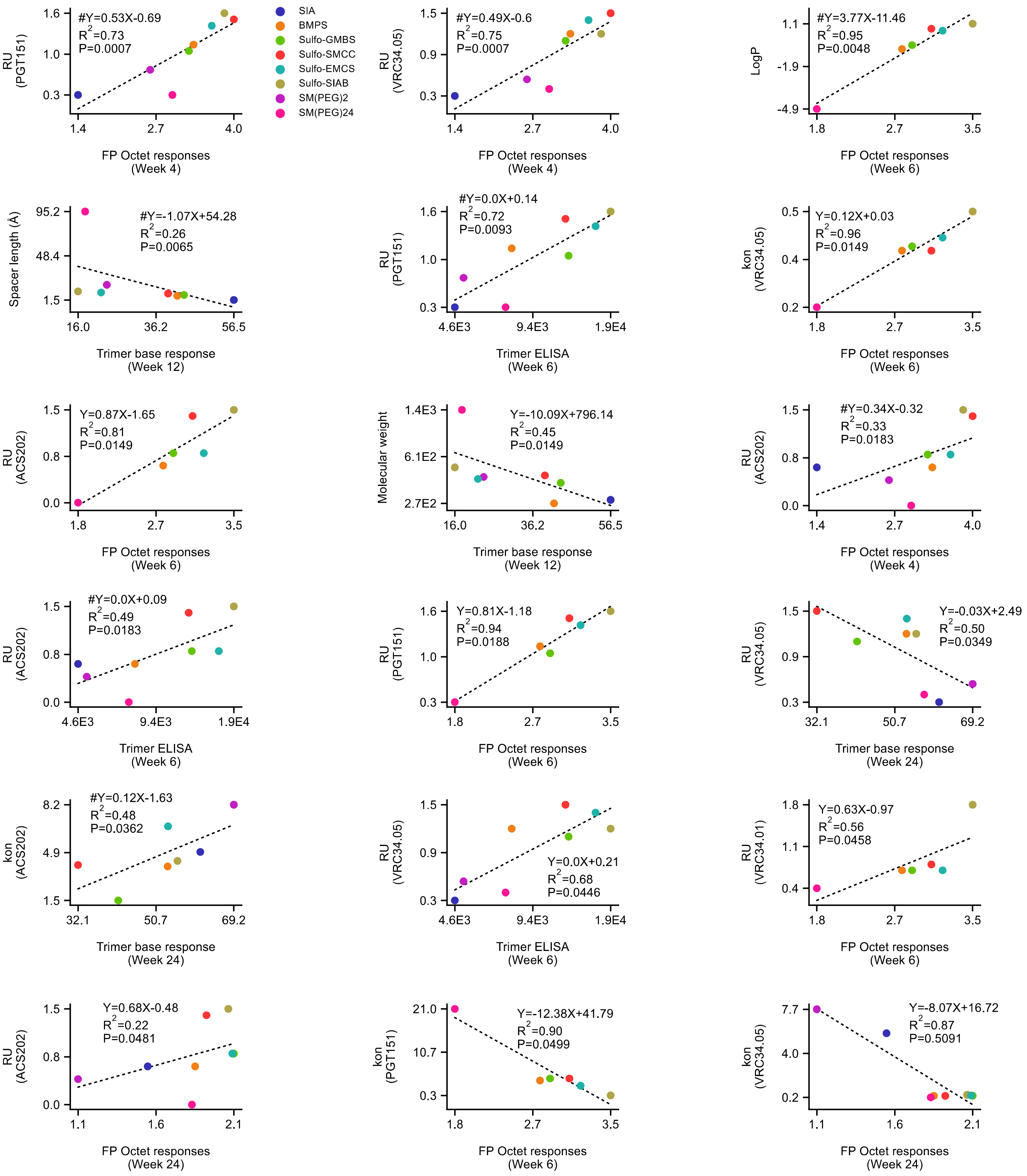

Figure S5. Visualizing correlations between linker and serological properties.
Correlation plots which 1) have high Spearman significance (p <= 0.05) or 2) fit to a linear (or log-transformed linear) model (R^2 >= 0.8) are shown, ordered left to right and top to bottom in terms of significance. Correlations which maintained Spearman significance when outlier SM(PEG)24 was excluded are denoted with a #. The linear (or log-transformed linear) regression model is also shown. Participant groups are colored according to linker, legend is shown.

Immunogen	Animal ID	BG505.W6M.C2.SG3 week 10	BG505.W6M.C2.SG3 week24	BG505.W6M.C2.N611Q.SG3 week 10	BG505.W6M.C2.N611Q.SG3 week24	MW965 week24	SIVmac251.30.SG3 week 10	SIVmac251.30.SG3 week24
Group1 SIA_FP8v1-rTTHC	N01	-43	35	-9	96	-15	-70	25
	N02	-24	31	91	98	30	-45	17
	N03	-30	3	57	85	2	-58	1
	N04	7	38	88	100	-3	-48	7
	N05	-3	35	42	78	-39	-25	14
	N06	-33	11	-7	83	-8	-35	-17
	N07	-14	36	22	68	-56	-54	-4
	N08	-13	56	-17	55	34	-41	-13
	N09	-2	37	74	75	14	-19	-5
	N10	1	50	85	77	-19	-50	-8
Group2 BMPS_FP8v1-rTTHC	N11	-37	69	29	98	43	-61	-15
	N12	-30	48	26	98	-18	-34	-2
	N13	-2	49	85	92	-4	-20	3
	N14	5	57	18	61	-42	-28	6
	N15	-30	-6	80	92	-18	-38	-13
	N16	-31	36	53	53	-2	-14	0
	N17	-53	27	95	98	-42	-43	-23
	N18	-48	44	-10	53	-57	-44	7
	N19	-32	13	37	55	-58	-26	-9
	N20	2	41	95	57	-4	2	7
Group3 Sulfo-GMBS_FP8v1-rTTHC	N21	-27	37	37	97	-43	-30	1
	N22	-33	25	20	85	-46	-40	-9
	N23	-21	93	71	97	-34	-23	8
	N24	-17	22	30	51	-57	-12	-5
	N25	-22	37	47	83	-45	-43	-3
	N26	-30	40	86	92	-9	-44	-5
	N27	-25	46	45	61	-67	-44	1
	N28	-30	83	60	99	-35	-18	7
	N29	-15	28	84	95	-27	-11	-4
	N30	-34	17	62	78	-62	-20	-13
Group4 Sulfo-SMCC_FP8v1-rTTHC	N31	-32	52	76	96	-51	-28	-9
	N32	-38	25	37	84	78	-23	-6
	N33	-41	43	70	79	-32	-43	-1
	N34	-49	64	16	95	-16	-33	0
	N35	-30	61	69	80	0	-47	-8
	N36	-19	55	27	90	8	-82	-6
	N37	-21	0	90	94	-15	-37	-5
	N38	-24	-4	9	78	-20	-64	-12
	N39	-22	18	30	92	-4	-43	-11
	N40	-19	51	68	64	-35	-52	-23
Group5 Sulfo-EMCS_FP8v1-rTTHC	N41	-2	37	90	97	-33	-71	-16
	N42	-33	18	50	88	17	-71	-24
	N43	-48	45	33	92	27	-67	-28
	N44	-23	32	84	96	-12	-66	-5
	N45	3	13	100	98	-12	-39	-47
	N46	-16	53	85	87	-13	-57	-9
	N47	-7	4	-4	48	-72	-51	-6
	N48	-15	49	72	96	24	-76	8
	N49	-23	22	35	95	-18	-72	0
	N50	-8	9	72	86	-51	-45	-1
Group6 Sulfo-SIAB_FP8v1-rTTHC	N51	-20	3	58	86	-74	-68	-24
	N52	-2	35	68	89	-43	-41	-9
	N53	10	43	90	80	-52	-37	-5
	N54	10	24	59	71	-26	-18	1
	N55	5	23	88	93	14	-48	-31
	N56	-18	-3	28	79	-84	-35	-21
	N57	-24	8	94	69	-58	-52	-21
	N58	-21	31	92	96	3	-62	-15
	N59	-13	10	17	68	-23	-44	5
	N60	-18	42	61	87	-4	-48	4
Group7 SM[PEG]24_FP8v1-rTTHC	N61	-5	53	-24	73	-23	-36	8
	N62	-12	7	84	87	-36	-47	-1
	N63	-1	5	-5	14	-36	-38	-25
	N64	8	48	1	53	-37	-30	6
	N65	-14	16	5	58	10	-62	-1
	N66	-33	-5	-52	6	-50	-35	1
	N67	-23	40	16	88	-39	-40	13
	N68	-50	10	-62	57	-32	-55	-4
	N69	-5	65	-1	88	-16	-35	-18
	N70	-22	17	-31	60	-35	-47	11
	VRC01	100		100		100		

0 100

Figure S6. Week 10 and 24 serum neutralization.
Virus neutralization assays with the immune sera at week 10 and 24. Sera at 1:50 dilution were used to neutralize the BG505 and its Δ611 virus.

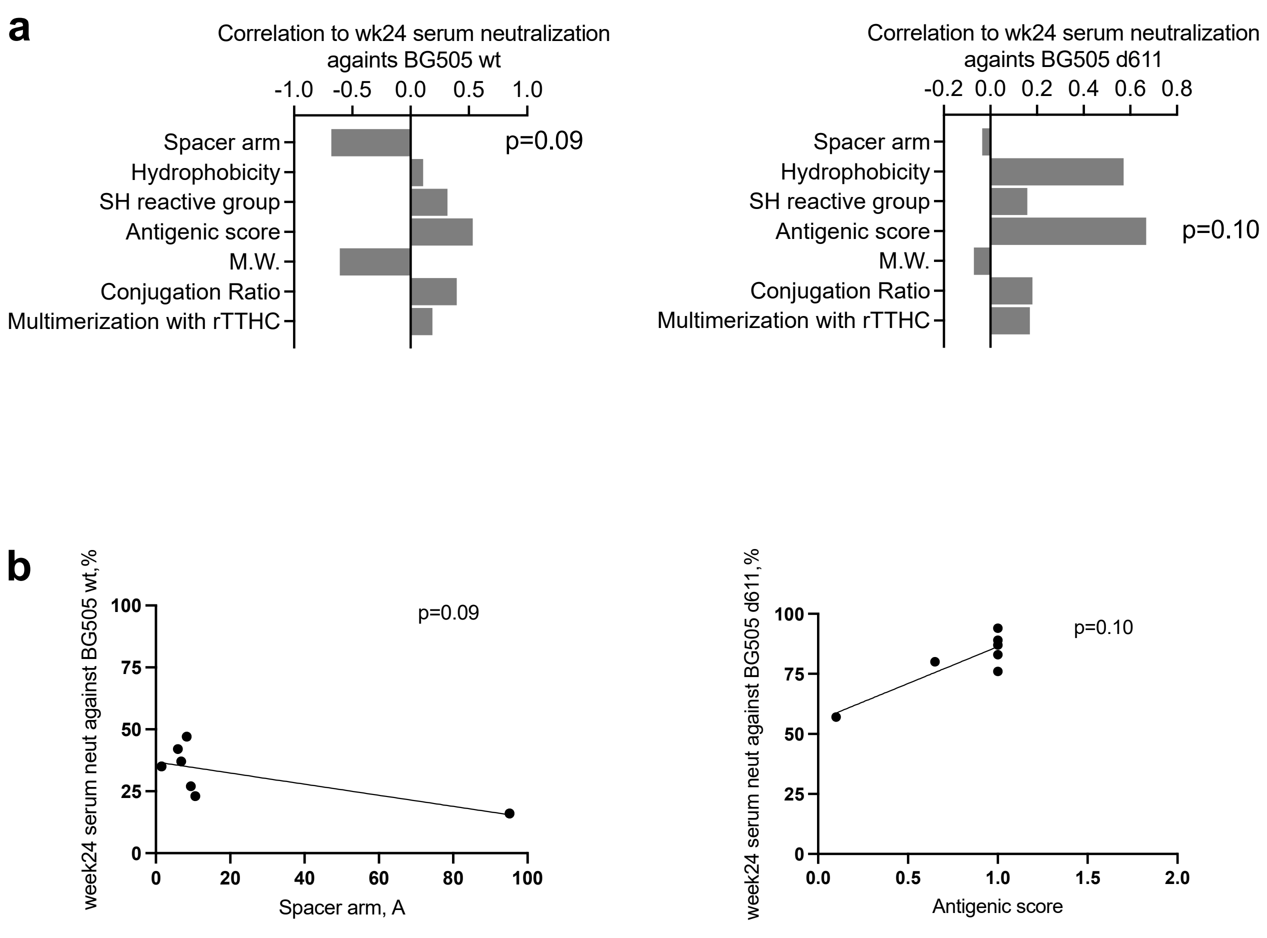

Figure S7. Correlation analyses between serum neutralization data and physical parameters of immunization.
(**a**) Virus neutralization assays with the immune sera at week 24. Sera at 1:50 dilution were used to neutralize the BG505 Δ611 virus. *P* values were calculated with Spearman correlation. (**b**) Plots for the largest correlations in panel a are shown, these correlation are not statistically significant, and correlation relative to the spacer arm are defined by a single outlier.

Neutralization

Linker and conjugate properties

Serological properties

	Neut. (Week 10, SG3)	Neut. (Week 10, N611Q.SG3)	Neut. (Week 24, SG3)	Neut. (Week 24, N611Q.SG3)
Spacer length (Å)	0.29	0.21#	-0.89#	0.00
Molecular weight	0.36	0.11	-0.82	0.00
Stoichiometry	-0.76	0.09	0.27	0.09
Multimerization	0.28	0.52	-0.22#	0.34
LogP	0.46	0.75	-0.11	0.68
SH reactive group	-0.79#	-0.16	0.00	0.16
Ag. score	-0.27	0.80	0.27	0.67
RU (VRC34.01)	0.04	0.82	-0.04	0.56
kon (VRC34.01)	-0.54	0.46	0.61	0.46
RU (VRC34.05)	-0.32	0.58	-0.20	0.68
kon (VRC34.05)	0.74#	0.45	0.31	0.32
RU (PGT151)	0.07	0.81	-0.27#	0.58
kon (PGT151)	-0.45	-0.70	0.09	-0.31
RU (ACS202)	0.24	0.87	-0.02	0.71
kon (ACS202)	0.60	0.14	-0.54	0.31
FP Octet responses (Week 4)	-0.14	0.68	-0.29	0.61
FP Octet responses (Week 6)	0.60#	0.94#	-0.09#	0.66
FP Octet responses (Week 24)	-0.04	0.82	0.14	0.71
Trimer ELISA (Week 6)	0.25	0.96#	-0.21	0.64
Trimer ELISA (Week 8)	-0.05	0.81	0.04	0.83#
Trimer ELISA (Week 10)	0.11	0.75	0.29	0.96#
Trimer ELISA (Week 24)	0.04	0.09	-0.41	-0.16
Trimer base response (Week 12)	-0.32	-0.36#	0.93#	0.00
Trimer base response (Week 24)	0.71	-0.36	-0.32	-0.54
Double positive B cells (%)	0.03	0.03	-0.37	-0.60

p value:
<= 0.0005
0.005 - 0.0005
0.05 - 0.005
> 0.05

R^2:
>= 0.8

Figure S8. Correlations between neutralization, linker, and serological properties.
Spearman rho values are shown. Correlations with high Spearman significance ($p <= 0.05$) are highlighted in purple (scale shown). Correlations which maintained Spearman significance when outlier SM(PEG)24 was excluded are denoted with a #. Correlations which failed to meet requirements for Spearman significance and Pearson's test assumptions but fit to a linear (or log-transformed linear) model ($R^2 >= 0.8$) are highlighted in red. For serological properties, the geometric mean was taken over each group, with 0 values converted to 1. For neutralization, a constant was added to all values, so all were >= 1 prior to geometric mean quantification. For the SH reactive linker property, Iodoacetyl was coded as 0 and Maleimide was coded as 1.

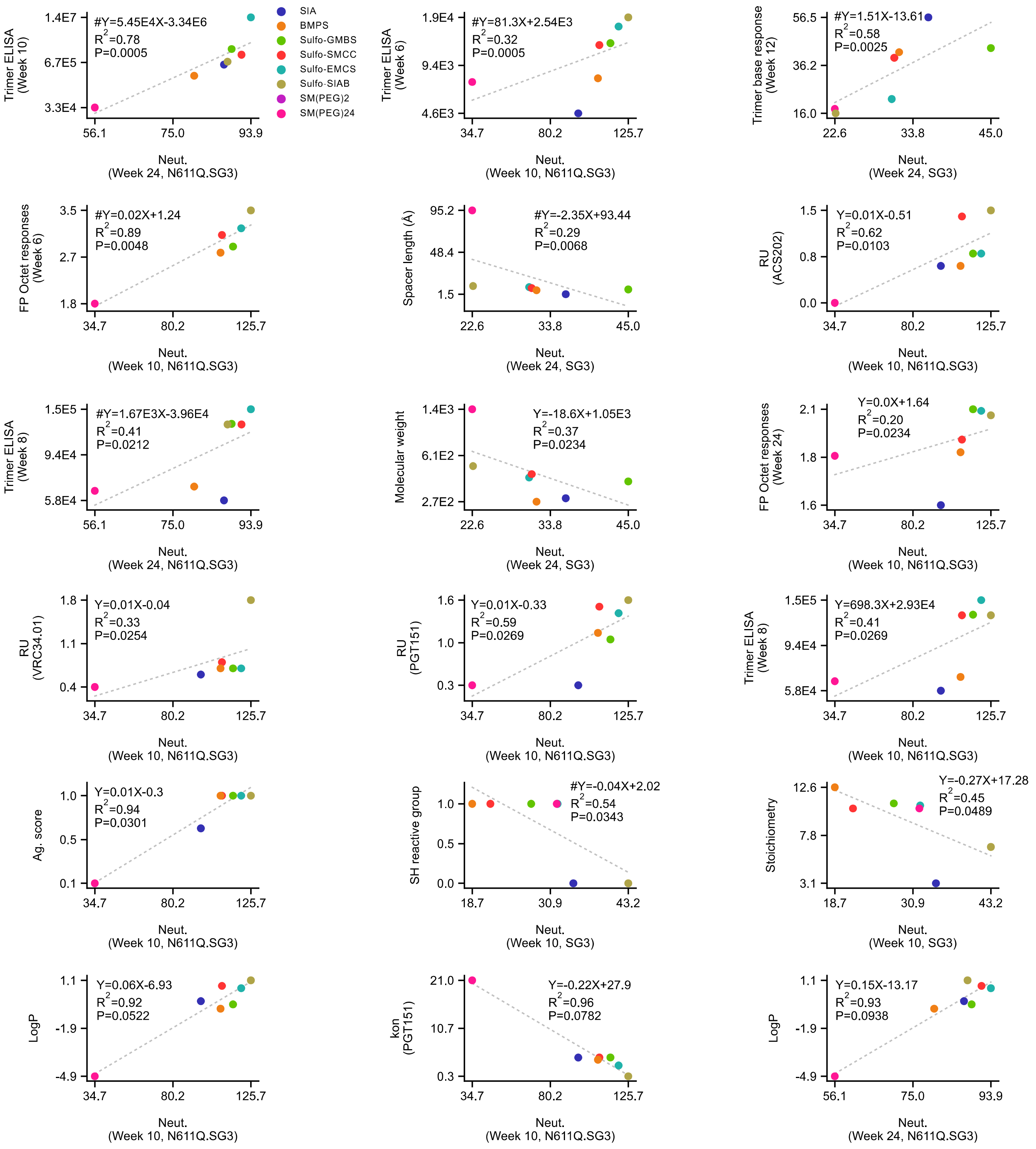

Figure S9. Visualizing correlations between neutralization, linker, and serological properties.
Correlation plots which 1) have high Spearman significance (p <= 0.05) or 2) fit to a linear (or log-transformed linear) model (R^2 >= 0.8) are shown, ordered left to right and top to bottom in terms of significance. Correlations which maintained Spearman significance when outlier SM(PEG)24 was excluded are denoted with a #. The linear (or log-transformed linear) regression model is also shown. Participant groups are colored according to linker, legend is shown.
